# Balance Training with Electromyogram-Triggered Functional Electrical Stimulation in the Rehabilitation of Stroke Patients

**DOI:** 10.3390/brainsci10020080

**Published:** 2020-02-02

**Authors:** Kyeongjin Lee

**Affiliations:** Department of Physical Therapy, College of Health Science, Kyungdong University, Gangwon-do 24764, Korea; kjlee@kduniv.ac.kr

**Keywords:** stroke, electric stimulation therapy, postural balance, rehabilitation

## Abstract

This study was conducted to investigate the effects of balance training with electromyogram-triggered functional electrical stimulation (EMG-triggered FES) to improve static balance, dynamic balance, and ankle muscle activation in stroke patients. Forty-nine participants (>6 months after stroke) were randomly assigned to the experimental group (*n* = 25) and the control group (*n* = 24). The experimental group underwent balance training with EMG-triggered FES for 40 min a day, 5 days a week, for a 6-week period in addition to general rehabilitation. The control group underwent balance training without EMG-triggered FES along with conventional therapy. Outcome measures included static balance ability, dynamic balance ability, and leg muscle activation. The static and dynamic balance abilities were significantly improved after intervention in both groups (*p* < 0.05), although the experimental group showed considerably greater improvement than the control group (*p* < 0.05). Leg muscle activation on the affected side resulted in significant improvements in the experimental group (*p* < 0.05) when compared with baseline but not in the control group. Balance training with EMG-triggered FES is an acceptable and effective intervention to improve the static balance, dynamic balance, and ankle muscle activation in stroke patients.

## 1. Introduction

Among the disorders caused by stroke, loss of balance and gait lead to significant inconvenience and result in difficulty with performing day-to-day activities [1,2]. Muscle weakness on the side affected by stroke causes restriction of movement, which in turn affects balance and walking ability [3,4,5]. Strength and balance, therefore, represent major challenges in the rehabilitation of patients affected by stroke [6,7].

In balance control for physical stability, the hips and ankles play important roles. Restoration of posture or maintenance of standing balance involves ankle or hip strategies [8]. The ankle joint strategy is the first postural control strategy that primarily restores standing balance through contractions of muscles involving the ankle joint when there is little fluctuation in solid support [9,10]. The alternating activation between the tibialis anterior and medial gastrocnemius keeps the balance during the anteroposterior sway. The activity of the medial gastrocnemius begins before the body collapses forward beyond the vertical line, and the activity of the tibialis anterior begins before the body leans back [11]. To control posture, muscles involved in moving the ankle joint not only alternate with concentric and eccentric contractions but also provide co-activation of the muscles in front of the knee and hip joints to provide stability [11].

Active, ongoing research has demonstrated that neuromuscular electrical stimulation has a positive effect on restoring lower limb motor function and on improving balance and gait [12,13]. Functional electrical stimulation (FES) is known for increasing muscle strength and for improving functional movements by stimulating the muscles which are not in voluntary control [14]. In studies applying FES to stroke patients with foot drop during walking, walking ability was improved by compensating for the lack of activation of ankle dorsiflexion during the swing phase [15]. In general, FES has been used to focus on these disorders during the swing phase within clinical settings. FES can correct swing-phase damage during walking; however, it is difficult to perform weight shift and weight bearing in the stance phase of the paretic limb [15,16]. FES can facilitate the walking motion using passive neuromuscular stimulation without active patient involvement. However, there is less convincing evidence that it improves balance and walking ability by inducing postural control in the ankle strategy.

Recent research has focused on active FES in contrast to traditional passive FES [17]. Electromyogram (EMG)-triggered FES initiates spontaneous contractions for specific movements until muscle activity reaches a preset threshold level, followed by secondary electrical stimulation [18]. Electrical stimulation that induces spontaneous contraction(s) is known to be more effective for muscle strengthening than passive FES. In existing applications of EMG-triggered FES, many studies have aimed to improve delicate hand manipulation and upper limb function in stroke patients [19,20]; however, investigating the effects of balance and gait intervention by ankle strategy were yet to be found. Therefore, the purpose of this study was to investigate the effect of EMG-triggered FES that induced an ankle strategy on static balance, dynamic balance, and leg muscle activation in patients affected by stroke.

The hypotheses of this study were as follows. First, there will be a difference in the amount of change on static balance ability in the pre-post by groups according to the intervention. Second, there will be a difference in the amount of change on dynamic balance ability in the pre-post by groups according to the intervention. Third, there will be a difference in the amount of change on leg muscle activation in the pre-post by groups according to the intervention.

## 2. Materials and Methods

### 2.1. Subjects

Stroke patients who were admitted to P Hospital in Gyeonggi Province, South Korea and were participating in rehabilitation programs were recruited for this study. The inclusion criteria were as follows: chronic hemiplegia ≥ 6 months due to stroke; Mini-Mental State Examination score ≥ 24; able to participate in exercise programs by understanding and executing verbal instructions; ability to stand unsupported for >30 min; and Brunnstrom motion recovery stage ≥ 4. Individuals with other abnormalities of the central and/or peripheral nervous system; with abnormalities of the musculoskeletal system including fractures; with visual or vestibular sensory disorders or cerebellar disease; and with unilateral neglect, apraxia, cardiovascular disease, or hearing impairments were excluded.

Subjects were given detailed information about the study including its purpose, procedures, and precautions before the study began. All subjects provided informed written consent to participate, and they provided their written informed consent prior to participation in accordance with the ethical principles of the Declaration of Helsinki.

### 2.2. Determination of the Sample Size

The study used a randomized controlled trial design, in which the assessors were blinded to clinical data. Alpha error and power probability were used to determine the sample size, with the alpha error set to 0.05 and the power set to 0.8. In addition, the effect size was set to 0.9 based on the velocity moment value in the static balance ability revealed in the pilot experiment. Therefore, 21 patients per group were required. Estimating a dropout rate of approximately 10%, 24 participants were required in a group. G* Power version 3.19 (Heinrich Heine University Düsseldorf, Düsseldorf, Germany), a statistical power analysis program, was used to calculate the sample size.

### 2.3. Procedure

**Figure 1:**
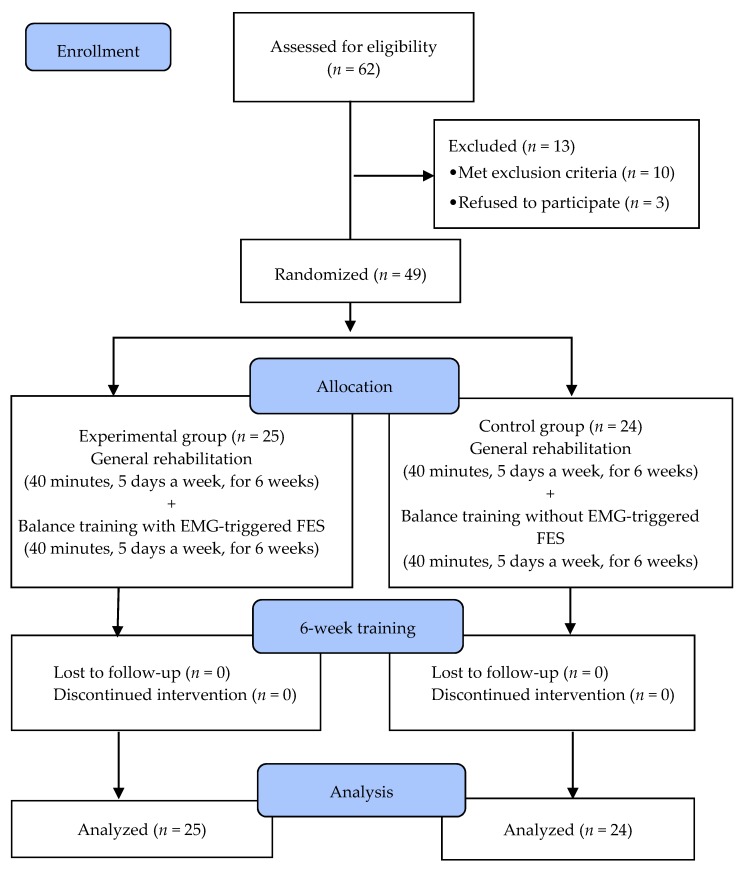
Flow diagram of the study.

In total, 62 chronic stroke patients were recruited for this study. Ten subjects were excluded as three of them had cognitive deficits, two displayed unilateral neglect, and five showed weakness in the lower extremities. Three additional subjects had to be excluded because they refused to participate. As such, 49 subjects were randomly assigned to the experimental (*n* = 25) or control (*n* = 24) groups. Random allocation software was used to minimize selection bias. All subjects underwent a 40-min rehabilitation training program five times per week over a six-week period. Subjects in the experimental group received 40 min of balance training five times per week using EMG-triggered FES (Figure 1). Subjects in the control group underwent 40 min of standing balance training five times per week without using EMG-triggered FES. To investigate the efficacy of the interventions, subjects were assessed for static postural balance, the Timed Up and Go test (TUG), the Berg Balance Scale (BBS), the Functional Reach Test (FRT), and leg muscle activation. These were evaluated one week before and one week after the six weeks of training. All evaluations were performed by two physical therapists who were blinded to the allocation of subjects to each training group.

### 2.4. EMG-Triggered FES

EMG-triggered FES treatment is an electrical stimulation method in which the triggered mode triggers low-frequency output and activates the muscle when the EMG signal is detected in the muscle. The equipment used in this study was performed with an EMG-triggered stimulation device (Stiwell med4, MED-EL, Innsbruck, Austria). This electrical stimulation device has four muscle stimulation channels and up to two EMG measuring channels. In the posture, various balanced exercises stimulated gastrocnemius and tibialis anterior related to the ankle strategy. The electrodes were attached to the lateral head of the gastrocnemius and the muscle belly of the tibialis anterior, and the measuring electrodes and the stimulating electrodes were attached at 1-cm intervals. The position of the electrode was attached by confirming activation in every training. All electrodes were square hydrogel electrodes (HRTS50AP-50 × 54 mm, Hurev, Wonju, South Korea). In order to determine the threshold of stimulation, the maximum activity of the two muscles was measured by moving the body from right to left and from front and back for 1 min in the standing position, and the initial threshold was determined when 70% of the maximum activity was applied. The threshold was adjusted according to the individual’s muscle contraction response during the training period. When the input stimulus reached the threshold, the stimulus was transmitted to the muscle through the FES electrode. Rectangular two-phase pulses with a pulse width of 300 μs were used. Pulse intensity (mA) was chosen to elicit sufficient contraction of the affected leg, depending on the target intensity (5–60 mA, average 16–18 mA). The standard current frequency is 30 to 35 Hz.

### 2.5. Balance Training Program

The balance training program was a modified version of a program described in a previous study [21]. In total, there were five phases of exercise, all of which were performed for 5 min each, with 2 min of rest, taking approximately 40 min, and with a total test time of 50 min. The balance training program was conducted five times per week under the supervision of a physiotherapist for six weeks. The six phases were as follows: (1) maintenance of a static posture; (2) maintenance of a standing posture with both feet; (3) maintenance of a forward and backward standing posture; (4) moving weight from left to right in a standing posture; and (5) maintenance of a static posture with plantar flexion and dorsiflexion.

### 2.6. General Rehabilitation

General rehabilitation was performed in both groups. This consisted of neurodevelopmental treatment and occupational therapy. Neurodevelopmental treatment was performed for 30 min with upper extremity exercise to not affect the experiment. Occupational therapy was 30 min in duration.

### 2.7. Outcome Measurements

A commercially available posturography system (GB300; Metitur Ltd., Jyvaskyla, Finland) was used as a method for measuring static balance ability. This system consists of a movable triangular platform for the feet, with a ruler marked on the platform for proper foot position, and is used to determine balance problems and the outcomes of rehabilitation; it is also used for training. This instrument has been widely used to measure balance in athletes, elderly individuals, stroke patients, and hemiplegics. In the test–retest method, the intraclass correlation coefficient (ICC = 0.83) in the measurer was proven to be higher than 0.83. The sampling frequency was 50 Hz.

The subject was standing three times, standing static on the equipment, with eyes open, and facing forward for 30 s. Three measurements were then taken, with the subjects’ eyes closed and standing forward for 30 s. 

The TUG was used to assess the dynamic balance ability of stroke patients. The TUG is a simple and quick test of functional movement consisting of getting up, walking 3 m, returning, and sitting. The method involves measuring the time it takes to get up from sitting in a chair with armrests and a backrest, to walk up to 3 m, and to then return to the chair. The test measures dynamic balance, functional movement, and walking ability in stroke patients with lower limb disorders such as stiffness. It is a highly reliable and valid method of evaluating risks (ICC = 0.99) [22]. In this experiment, 50-cm tall chairs were used for measurement; the measurer performed three measurements using a stopwatch and calculated and recorded the average value.

The FRT is used to assess the limits of stability. In this measurement method, the subject stands at a distance of approximately 10 cm from a wall, bends their shoulders 90° while holding fists, and extends their arms as far forward as possible in a direction parallel to the floor. The assessment–reassessment reliability and inter-measurement reliability are high, r = 0.89 and r = 0.98, respectively [23]. Three measurements were performed in this experiment to calculate and record the average value.

The BBS is used to measure the balance ability of stroke patients or of elderly individuals. The 14 items were scored on a 5-point scale from 0 to 4, with a total score of 56 [24]. This instrument has high reliability and internal validity in evaluating balance ability (r = 0.99 and r = 0.98, respectively) [25].

Surface EMG (Telemyo DTS, Noraxon Inc, Scottsdale, AZ, USA) was used to measure muscle activity. Surface EMG amplifies the voltage that occurs when muscles contract. Then, the voltage is filtered and sent to the receiver for signal acquisition. The collected data was analyzed using EMG software (myoRESEARCH MR 3.5.4, Noraxon Inc, Scottsdale, AZ, USA).

Each participant wore the same patient suit and personal shoes. To cleanse the skin over the target muscle, alcohol and skin prep gel were used. Two disposable EMG electrodes with a 2.0-cm interelectrode distance were attached to the tibia anterior and lateral heads of the gastrocnemius muscle. Muscle activity was measured both on the affected and unaffected sides. Following surface electromyography for the non-invasive assessment of muscles (SENIAM) guidelines, the tibialis anterior electrode was located one third of the line between the end of the fibula and the end of the medial cartilage, while the gastrocnemius electrode was located one third of the line between the head and heel of the fibula. The position of the electrodes is marked with a pen to measure at the same position.

Subjects underwent maximal voluntary contraction (MVC) for passive resistance and recorded EMG activity from each of the two muscles. All measurements were made three times, measured for 5 s, and analyzed for values obtained in the middle 3 s. For MVC examination of tibialis anterior, the subject was in the sitting position and the therapist fixed the patient’s ankle, resisted the instep with one hand, and induced maximum spontaneous contraction. For gastrocnemius MVC examination, the subject was in the sitting position and the therapist fixed the patient’s ankle, resisted the sole with one hand, and induced maximal voluntary contraction. The sampling rate of the measured data was set to 1000 Hz, and the bandwidth was set to 20–400 Hz. The measured EMG signal was processed using the root mean square (RMS) value after rectification, and the average value of the three measured values was used.

### 2.8. Statistical Analysis

All data are expressed as mean and standard deviation. The Shapiro–Wilk test was used to test data normality; all variables were found to satisfy the normality assumption. The paired *t*-test was used to compare the dependent variables within groups, and the independent *t*-test and chi-squared test were used to compare the dependent variables between the two groups. Differences with *p* < 0.05 were considered statistically significant. SPSS version 20.0 (IBM Corporation, Armonk, NY, USA) was used for statistical analysis.

## 3. Results

In all, 49 participants in both groups completed 6 weeks of intervention therapy and associated assessments. The general characteristics of the participants are shown in Table 1. No significant difference was observed in the baseline values for the general characteristics or dependent variables of the two groups.

Outcome measures of static balance ability, dynamic balance ability, and leg muscle activation of the experimental and control groups are shown in Table 2. The changes in static balance ability, the speed of medial and lateral sway, the speed of anterior and posterior sway, and the velocity of moment variables showed significant improvement after intervention in both groups regardless of eyes-open or eyes-closed states (*p* < 0.05). However, the experimental group showed a greater improvement than the control group (*p* < 0.05). Dynamic balance ability determined using TUG, BBS, and FRT showed significant improvement after intervention in both groups (*p* < 0.05). However, the experimental group showed significantly more improvement as compared to the control group (*p* < 0.05). The changes in muscle activation of the tibialis anterior and gastrocnemius on the affected side showed a significant improvement after intervention in the experimental group (*p* < 0.05) but not in the control group. The leg muscle activation on the unaffected side was not significantly changed after intervention in both groups.

## 4. Discussion

The purpose of this study was to evaluate the effects of balance training with EMG-triggered FES on the balance and ankle muscle activation of chronic stroke patients. EMG-triggered FES is used to induce spontaneous contraction and subsequent stimulation of the muscles by providing an electrical stimulus. In contrast to previous studies aimed at improving the gait, this study identified the effect of electrical stimulus on balance ability and leg muscle activation during balance training. The experimental group improved both their static and dynamic balances more than the control group. Stroke patients live with sequelae due to physical impairment caused by damages to the motor and sensory areas of the cerebral cortex [2]. Balance abnormality is a typical symptom, and improving balance is one of the important goals of stroke rehabilitation [7]. Static balance ability improves both the anterior–posterior and medial–lateral balance either with or without visual block.

Electrical stimulation has been studied previously for stroke rehabilitation whereby FES was applied during the swing phase of walking to activate nerves that stimulate the lower extremity affected by stroke [12,13,14,15,16]. Many previous studies have observed the positive effect of FES on the gait ability of stroke patients [15,16,26]. However, some studies have reported that, although FES can improve the walking ability, there are no additional benefits for motor functions [15,16]. Without active participation of the subject, preprogrammed FES stimulation could only cause periodic muscle contraction. In particular, previous studies have shown that active participation in exercise training is more effective than passive participation in improving functional movement [26,27].

Triggered FES is provided when a spontaneously generated muscle contraction signal exceeds a preset threshold. Triggered FES had a positive effect not only on the movement and function of the paralyzed arm in stroke patients but also on walking [19,20,27]. Triggered FES when combined with voluntary efforts is likely to increase functional movement [19,28]. The results of the leg muscle activation show that there is an improvement in the affected side, and the improved results of the experimental group using EMG-triggered FES means that the functional movement of stroke patients is improved by facilitating retraining exercises with triggered FES.

Dynamic balance was assessed using BBS, TUG, and FRT; the experimental group showed a significant improvement in dynamic balance. The results of this study indicate that training combined with electrical stimulation in the affected leg is an effective intervention for improving balance.

The TUG is a tool that measures the ability to get up, to walk, to turn, and to sit on a chair. If it takes more than 14 seconds, the subject is considered to have a high risk of falling [29]. Stroke patients appear to take times varying between 20 and 50 seconds depending on the severity of the disorder [30,31]. In this study, the average TUG of the two groups was about 30 seconds, whereas in other related studies with stroke, patients had a milder level. In the Iruthayarajah et al. [30] study, virtual reality training reported that the minimal detectable change (MDC) score was 4–9 s in a system review of the study applied to stroke patients.

The BBS is a dynamic balance measure that assesses the balance and assesses the risk of falls. Forty-one to 56 points are low fall risk, 21–40 points are medium fall risk, and 0–20 points are high fall risk. In this study, the experimental group showed a clinically significant decrease of 4.7 s. In a recent study of the elderly, when the patient’s BBS scored was 45–56 points, the MDC score was 4 points; at 35–44 points, the MDC score was 5 points; and at 25–34 points, the MDC score was 7 points. Finally, MDC was 7 when the score was 25–34. In the Iruthayarajah et al. [30] study, virtual reality training reported that the MDC was 4–6 points through a system review of the study applied to stroke patients. In the present study, the experimental group showed an increase of 1.40 points, indicating a slight change. Though considered a statistically significant improvement, it is not enough to eliminate the risk of falling since it showed a significant improvement in comparison with the control group.

The FRT was developed to predict “falls” in older people. If the reach is less than 15 cm (6 inches), it is considered fall risk and weakness. In the Katz-Leurer et al. [23] study, virtual reality training reported that the MDC of the paretic side was 2.3 cm through a system review of the study applied to stroke patients. In this study, the experimental group showed an increase of 3.7 cm, which is clinically significant.

People use ankle or hip strategies to balance the lower extremities. Narrow stance width increases ankle angular movements, hip angular movements, and medial–lateral sway of the center of gravity (COG). The ankle mechanism is dominant during normal stance, especially in the sagittal plane. The gastrocnemius and tibialis anterior are key muscles in the control of the sagittal movement of the ankle [8,9,10]. In this study, EMG-triggered FES was applied to tibialis anterior and gastrocnemius, which are important muscles for the ankle strategy. We used EMG to identify changes in muscle activation before and after intervention. The control group showed no significant difference between the affected and unaffected sides whereas the experimental group showed a substantial increase in muscle activation primarily on the affected side. This suggests that FES consistently stimulates ankle muscles during balance training, constricting dorsiflexor and plantar flexor in the ankles, leading to increased muscle activation.

The time-delay seemed to have occurred while applying electrical stimulation to the target muscle fiber. Unfortunately, the exact delay time could not be measured in this study. In future studies, using force plates or motion analysis to contract muscles with accurate timing would be a more effective training method. Although this study was conducted in patients with chronic stroke, it is expected to be more effective when applied to acute patients; we hope to confirm this effect in future studies. Our study could only validate the immediate effect of the intervention but could not observe how long it lasted. A follow-up study is thus necessary to identify long-term effects.

## 5. Conclusions

Balance exercise combined with EMG-triggered FES is a useful rehabilitation program for restoring the static and dynamic balances of stroke patients, and it has been shown to improve the activity of the ankle muscle. Our findings suggest that balanced training combined with EMG-triggered FES can help improve functional movement in stroke patients.

## Figures and Tables

**Table 1:** General characteristics of the subjects.

	Experimental Group (*n* = 25)	Control Group (*n* = 24)	χ^2^/*t*	*p*
Age (year)	62.16 ± 8.13	64.88 ± 10.35	1.023	0.311
Height (cm)	163.36 ± 8.80	164.88 ± 7.86	0.634	0.529
Weight (kg)	63.14 ± 6.37	65.42 ± 8.07	1.099	0.278
BMI (point)	23.79 ± 3.15	24.05 ± 2.32	0.322	0.749
Time since stroke (month)	16.00 ± 6.49	15.25 ± 6.89	0.392	0.697
MMSE	25.92 ± 1.22	25.75 ± 0.94	0.543	0.589
Gender (male/female)	17/8	15/9	0.686	0.163
Paretic side (right/left)	16/9	14/10	0.684	0.166
Stroke type (infarction/hemorrhage)	17/8	13/11	0.686	0.163

Values are expressed as mean ± standard deviation. The independent *t*-test and Chi-squared test are used to compare the dependent variables between the two groups. BMI, body mass index; MMSE, mini-mental state examination.

**Table 2:** Comparison of measures within groups and between groups.

Variables	Experimental Group (*n* = 25)			Control Group (*n* = 24)			Significance of Change Scores
	Baseline	Post	Change Score	Baseline	Post	Change Score	t
Static balance ability							
EO-MLS (mm/s)	4.04 ± 1.36	2.95 ± 0.69	−1.08 ± 1.01 *	3.68 ± 1.16	3.19 ± 0.88	−0.49 ± 0.83 *	2.225 ^†^
EO-APS (mm/s)	5.97 ± 1.55	4.74 ± 1.52	−1.23 ± 0.75 *	5.81 ± 1.14	5.10 ± 1.25	−0.71 ± 0.63 *	2.614 ^†^
EO-VM (mm/s^2^)	4.32 ± 1.47	2.84 ± 1.47	−1.47 ± 0.73 *	3.96 ± 1.08	3.18 ± 1.24	−0.78 ± 1.02 *	2.737 ^†^
EC-MLS (mm/s)	4.35 ± 1.45	3.21 ± 0.96	−1.13 ± 0.79 *	4.39 ± 1.17	3.87 ± 1.38	−0.52 ± 0.70 *	2.870 ^†^
EC-APS (mm/s)	6.05 ± 1.55	5.12 ± 1.50	−0.93 ± 0.75 *	5.61 ± 1.14	5.14 ± 1.04	−0.47 ± 0.63 *	2.700 ^†^
EC-VM (mm/s^2^)	5.12 ± 2.08	3.69 ± 1.58	−1.43 ± 0.98 *	4.63 ± 2.02	4.06 ± 1.97	−0.57 ± 1.03 *	2.989 ^†^
Dynamic balance ability							
TUG (sec)	31.56 ± 7.12	29.41 ± 6.25	−2.15 ± 2.39 *	29.53 ±3.89	28.68 ±4.14	−0.85 ± 1.70 *	2.193 ^†^
BBS (point)	44.48 ± 3.44	45.88 ± 4.10	1.40 ± 1.53 *	43.38 ± 3.52	43.83 ± 3.40	0.46 ± 0.93 *	2.592 ^†^
FRT (mm)	189.99 ± 54.35	222.24 ± 57.13	32.24 ± 24.56 *	192.32 ± 73.28	203.49 ± 78.48	11.17 ± 17.39 *	3.454 ^†^
Leg muscle activation							
Tibialis anterior affected (µV)	5.59 ± 3.69	10.79 ± 3.99	5.20 ± 1.30 *	6.93 ± 2.17	7.72 ± 2.60	0.80 ± 1.80	9.693 ^†^
Tibialis anterior unaffected (µV)	33.77 ± 15.20	32.64 ± 14.67	−1.13 ± 3.67	31.16 ± 16.91	32.09 ± 15.76	0.93 ± 3.07	2.121 ^†^
Gastrocnemius affected (µV)	11.99 ± 8.29	19.11 ± 8.35	7.12 ± 1.32 *	11.92 ± 5.81	9.98 ± 5.19	−1.94 ± 8.21	5.446 ^†^
Gastrocnemius unaffected (µV)	59.46 ± 40.63	58.75 ± 40.62	−0.71 ± 2.19	64.78 ± 28.06	64.91 ± 27.95	0.13 ± 1.31	1.623 ^†^

Values are expressed as mean ± standard deviation. * Significant difference within group. ^†^ Significant difference between group. EO, eye opened; EC, eye closed; MLS, medial–lateral speed; APS, anterior–posterior speed; VM, velocity moment; TUG, Timed Up and Go test; BBS, Berg Balance Scale; FRT, Functional Reach Test.
